# Growth model analysis of wild hyacinth macaw (*Anodorhynchus hyacinthinus*) nestlings based on long-term monitoring in the Brazilian Pantanal

**DOI:** 10.1038/s41598-022-19677-5

**Published:** 2022-09-13

**Authors:** Neiva Maria Robaldo Guedes, Maria Cecília Barbosa Toledo, Fernanda Mussi Fontoura, Grace Ferreira da Silva, Reginaldo José Donatelli

**Affiliations:** 1 Environment and Regional Development Graduate Program-Anhanguera, Uniderp University, Campo Grande, MS Brazil; 2 Instituto Arara Azul-ITA, Rua Klaus Sthurk, n. 106, Jardim Mansur, Campo Grande, MS 79.051-660 Brazil; 3 Environmental Science Graduate Program, Universidade de Taubaté-UNITAU, Rua Visconde Do Rio Branco 210, Taubaté, SP 12.020-040 Brazil; 4 Zoology Graduate Program, Universidade Estudual Paulista “Júlio de Mesquita Filho”-UNESP, Av. Eng. Luiz Edmundo C. Coube 14-01-Núcleo Habitacional Presidente Geisel, Bauru, SP 17033-360 Brazil

**Keywords:** Biological techniques, Ecology, Zoology, Ecology, Environmental sciences

## Abstract

Studies on the breeding of vulnerable and endangered bird species are hindered by low numbers of individuals, inaccessible location of nests, unfavourable environmental conditions, and complex behavioural patterns. In addition, intraspecific variation may emerge only following long-term, systematic observations of little-known patterns and processes. Here, data collected over 30 years were used to determine growth model of hyacinth macaw (*Anodorhynchus hyacinthinus*) chicks in the Pantanal biome of Brazil. During this period, the speed of growth and body mass of chicks varied widely. Four growth models were tested: logistic, Gompertz, Richards, and cubic polynomial. They were fitted using three biometric measurements: body mass, total length, and tail length. The best-fitting growth curves were identified using Akaike’s information criterion. The best models were the cubic polynomial for body mass, Richards for total length, and Gompertz for tail length. We confirmed the occurrence of dwarf individuals, whose body mass, total length, and tail length were 20%, 22%, and 70% smaller, respectively, than in the overall population. The dwarfs remain small in size after having fledged and are easily identified as adults. We discuss the importance of long-term studies to identify windows of opportunity for further research that will help in the conservation of endangered macaw species.

## Introduction

During the last 20 years, many species of Psittaciformes have faced an increasingly dire scenario, with an estimated 42% of them being endangered or near-threatened^1^. Berkunsky et al.^2^ identified agriculture, pet trade, logging, and human intrusions and disturbance among the various factors threatening neotropical parrots. Of the 86 known Brazilian parrot species, 17 are threatened nationwide^3^, two are extinct^4,5^, and 25 are threatened at a global level^6^.

The rapid and relentless loss of habitat, as well as the trade in wild animals have a particularly strong impact on parrots such as hyacinth macaws (*Anodorhynchus hyacinthinus*) characterised by a slow reproductive cycle^7,8^. The ongoing Arara Azul Project, which aims to improve the reproductive fitness of this species which is vulnerable to extinction^3^, has managed to boost the number of individuals, from 2500 in 1980 to 6000 in 2018^8–11^. However, during 2019 and 2020, new threats have arisen, such as poisoning by agrochemicals^12^ and intense fires, which have substantially increased the mortality rate among nestlings and young individuals in the Pantanal region of Brazil^13,14^. Moreover, anthropogenic interventions, such as deforestation and fires, have drastically affected the population dynamics of the acuri palm (*Scheelea phalerata*), which constitutes the main food source for the hyacinth macaw during breeding^15^. During this period, marked differences have been observed in the size of some nestlings from the same clutch, with one nestling being smaller than the sibling and the population average.

The hyacinth macaw is an altricial species, which remains in the nest and is dependent on its parents for approximately three and a half months^7^. During this period, numerous factors can influence the growth of nestlings^7,10,16,17^. A number of factors have been found to directly affect the growth rate of Psittacidae, including environmental parameters (e.g. temperature and precipitation), food quality and availability, differences between the sexes, hatching-rank effects, clutch size, diseases, and ectoparasite infestations^8,10,13,14,18–22^. As a result, these factors generate intraspecific variations can lead to significant differences in growth rate, which in turn, can favourite adaptive processes such as a decreased competition between nestlings of the same clutch, or negative ones such as a reduced fertility^10,16,18^.

Given the many variables affecting Psittacidae fitness in the wild, growth models based on data collected from natural populations are tools which are important to help in the conservation of vulnerable species or those threatened with extinction. Growth models based on long-term datasets detail intraspecific variation that can aid in decision making regarding: (1) management of projects conducted in the field and aimed at invigorating wild populations; (2) rehabilitation and reintroduction to nature of specimens apprehended from illegal trade and the voluntary surrender of individuals rescued in natural environments; (3) management of pairs that occupy altered and fragmented habitats; and (4) raising specimens in captivity^19,23,24^. Additionally, growth curves can aid in the monitoring of populations that are managed in the field; these populations are a good indicator of the health of the species as well as of its environment^25^. Here, our objective was to analyse the growth of nestlings from a natural population of hyacinth macaws over 30 reproductive periods in the Brazilian Pantanal. To this end, different growth models were compared, and individual variations were fitted into the models to identify the most accurate ones.

## Material and methods

### Study area

**Figure 1:**
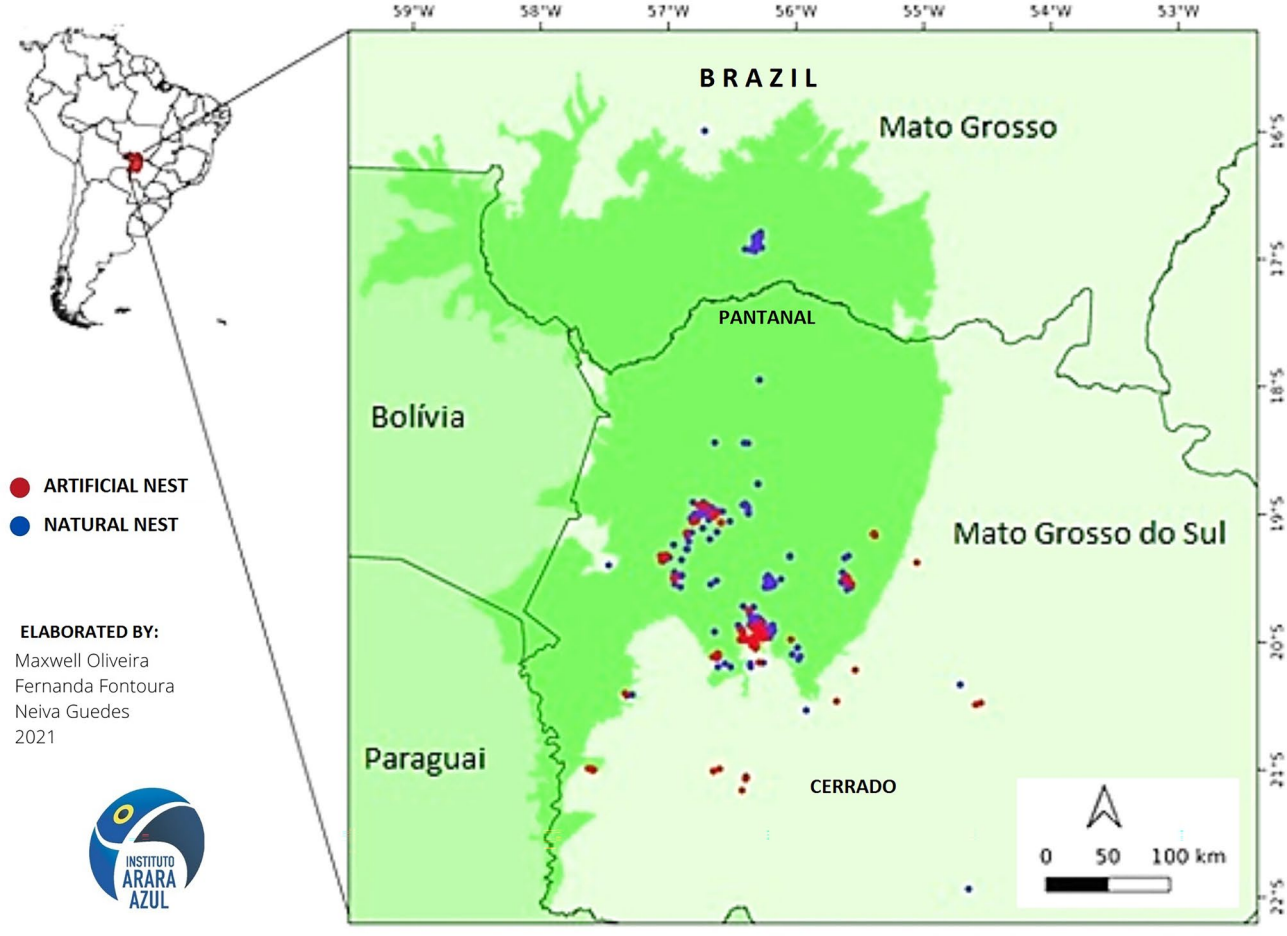
From 1991 to 2021, natural nests (n = 473, in blue) and installed artificial nests (n = 415, in red) were monitored in the Pantanal in the states of Mato Grosso do Sul and Mato Grosso, and more recently (last 15 years) in the Cerrado biome. This map was prepared independently with qgis v 3.10 software (www.qgis.org). Source of political boundaries: IBGE (www.ibge.gov.br).

During a period of 30 years (1991–2021) fieldwork were performed in the Pantanal region spanning the Brazilian states of Mato Grosso do Sul and Mato Grosso (16° 21′ S and 55° 58′ W) and in the last 15 years, in the Cerrado biome (Fig. 1), comprising a total area of approximately 3000 km^2^. Located in the states of Mato Grosso do Sul and Mato Grosso, but extending also into Bolivia and Paraguay, the Pantanal is the largest continental wetland area on the planet, covering approximately 140,000 km^2^^26,27^. The climate of the Pantanal is classified as tropical wet according to the Köppen classification system and has an annual average temperature of 25 °C. Rainfall is more pronounced between November and April, resulting in two relatively well-defined seasons: a dry winter and a rainy summer season. According to Keuroghlian and Desbiez^28^, annual precipitation varies between 600 and 1700 mm (average of 1250 mm) in flooded areas, but is generally higher in upland areas, with an average of 1500 mm. The landscape of the Pantanal is a direct result of hydrological cycles. During the rainy season, up to 80% of the region is flooded, which makes access to this area difficult, and has discouraged human settlement. The complex hydrological network and large seasonal variations in water level have created a nutrient-rich environment that offers shelter and food to a diverse and abundant fauna^29,30^. Due to long periods of inundation, difficulty of access, and low population density, the Pantanal remains largely intact and is one of the most preserved biomes in Brazil, to the point of being designated a UNESCO Biosphere Reserve and a World Heritage Site. However, in recent decades this biome have suffered with expansion and intensification of croplands, livestock, mining, and large government infrastructure projects^31^. These activities have greatly altered the Pantanal by accelerating deforestation and uncontrolled fires^8,13,26,31,32^, which has had negative repercussions on sensitive or threatened species, causing a loss in biodiversity^14^.

In order to develop the research, we received permission from the owners to enter the ranches in the Pantanal, follow the hyacinth macaws and monitor their nests. The fieldwork began before the formation of the Chico Mendes Institute of Biodiversity Conservation, ICMBio, in the year 2007, which currently authorizes and regulates scientific research in the country. At the time when this study begun, there was also not any need of a requirement to submit projects to ethics committees for studies on wildlife. NMRG (first author) was part of the hyacinth macaw conservation committees established by the Federal Government of Brazil since 1999, according to Ordinance No. 59 of the Brazilian Institute of Environment and Natural Resources, published on July 15, 1999.

### Breeding characteristics

The hyacinth macaw is a species that is non migratory, monogamous, and highly specialized with respect to its feeding and nesting sites. Ninety-five percent of the nests are built in hollows in the manduvi tree (*Sterculia apetala*). There is strong competition among breeding hyacinth macaw pairs and other species for these hollows because only trees older than 60 years produce cavities large enough to be used by the macaws^33^. Approximately 75% of females lay two eggs, and of these there is a probability of 53% to lose one egg^7,10^. According to Guedes, for every 100 eggs laid, only 25% of them will survive until the chick leaves the nest^10^. The hyacinth macaw is an altricial species^34^, that is does not finish growing during the period it remains in the nest, that is, it only reaches the size of an adult after leaving the nest.

### Field protocol

**Figure 2:**
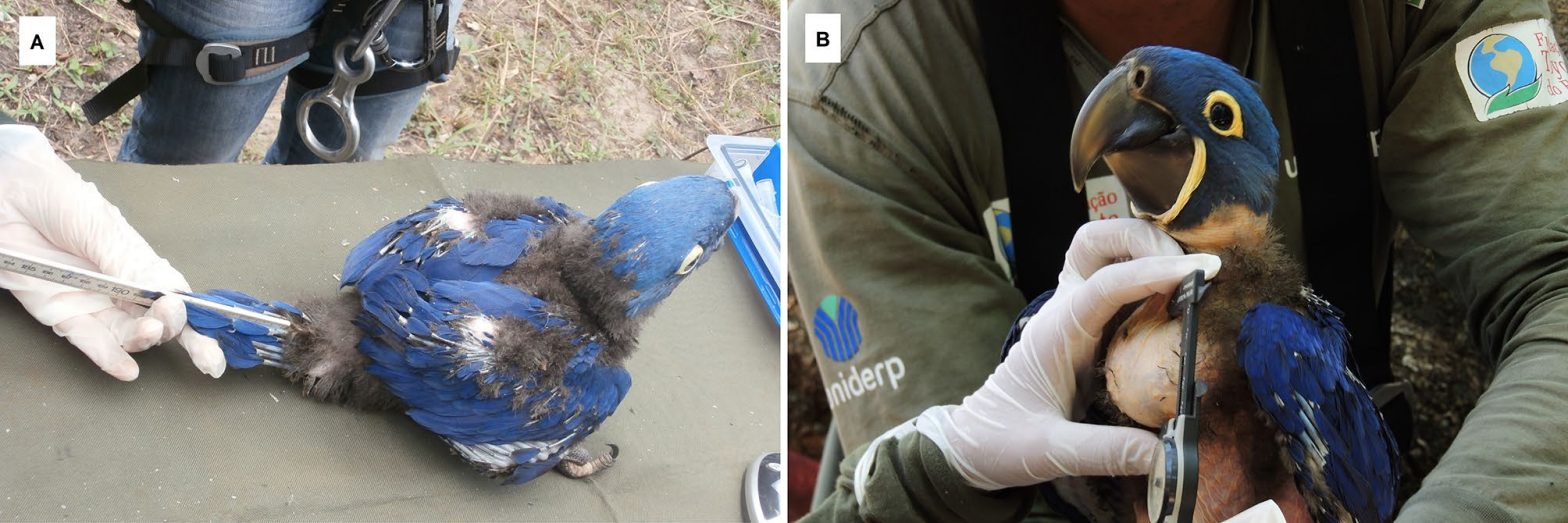
Measurements of the tail length (**A**) and crop (**B**), and nestling mass for the hyacinth macaw in the Pantanal.

The process was initiated by observing a hyacinth macaw pair defending the nesting cavity in a tree. Once egg laying was documented, the nest was inspected regularly until the fledging of the chick, with day 1 defined as the day the egg hatched^7,10^. An OHAUS digital scale with a capacity of 2 kg was used to determine weight (g). Total length (mm) and medial tail feather length (mm) was measured with a metal ruler to the nearest 1 mm (Fig. 2A). To minimize potential observer bias, the measurements were conducted by the same person during the first 15 years and, thereafter, by two other researchers who rigorously adhered to a training protocol. Due to the location of the nests, difficulty to walk in the study area, and floods during certain periods of the year, the chicks were weighed at different day and time intervals. Additional weight variation came from variation of the quantity of food in the crop. The diameter of the crop was measured using a digital caliper to nearest 0.01 mm, and the volume (V) was calculated as V = 4*π*r^3^/3 (Fig. 2B). A model made of latex film and having the same volume as the crop when completely filled with *Scheelea phalerata* (Mart ex Spreng) Burret (Arecaceae) was constructed in the laboratory and the density (d = mass/volume) was calculated. In this way, the mass (M) of the crop was individually calculated as M = d*V and subtracted from the total body mass of the nestlings.

Hyacinth macaws are not sexually dimorphic, therefore, sex was identified by a blood sample of 0.1 ml from the brachial wing vein. The samples were kept at room temperature in absolute ethanol and sent to the Genetics and Molecular Evolution Laboratory at the Biology Institute of the University of São Paulo, Brazil. In the laboratory, DNA was extracted and PCR was run for sex identification following Miyaki et al.^35,36^. Due to logistical challenges in sending the samples, as well as to the remoteness of the nests and unfavorable weather conditions, not all the chicks were sexed (sex identification method details see Miyaki et al.^36^).

Of the 837 nestlings, not all individuals measured fledged; 395 died, mainly due to predators or other natural causes, such as infestation by ectoparasites. From more accessible nests, 42 eggs and from hatching (day 1) 30 nestlings (12 eggs failed) were monitored at the same time of day and with high-resolution temporal sampling (55 ± 11 days). The others 412 individuals were measured, from day 1, at the different time of day and with low-resolution temporal sampling (12 ± 7 days). Therefore, we included in this study only the individuals measured and weighed until they developed their full plumage. During the fieldwork we had already identified a group of nestling with metrics well below the other individuals, which we termed “dwarfs”. To identify and isolate the dwarfs from the total study population of 412 nestlings, we used field observations and cluster analysis (Euclidean distance and Ward methods) for the three metrics of body mass, total length and tail length. Cases of dwarfism were observed in the first and second nestlings for males and females. The total number of dwarfs observed was 31 individuals over 30 years of fieldwork; however, only 15 entered the analysis, which were those individuals that fledged. For a better flow of text, we termed the other individuals “normal-sized” birds.

### Statistical analyses

We performed 89 measurements of the 15 fledged dwarfs and 1119 measurements of the 381 fledged normal-sized birds. Asymptotic models commonly used for the analysis of bird growth, including the Gompertz, Richards, and logistic models (sigmoidal curves), were evaluated, whereas a cubic polynomial model was applied to more complex growth curves. All models were fitted by minimising sum-of-squares of the residual errors and no parameters were constrained. In order to select the best model, we used the Akaike information criterion (AIC) values for sample sizes of > 30 individuals and AICc values for sample sizes of < 30 individuals, the *F* test, standard error of the residuals and selected the one that visually best fitted the data. Unlike the Akaike's criterion, which selects the model with highest probability to be correct^37^; the *F* Test defines the best model as the one with the lowest sum-of-squares. Using the best models for each of the morphological traits, we analysed changes to different growth curve parameters by including the upper asymptote (A), maximum growth rate (*k*), initial value (Y0) of the asymptotic curve for the Gompertz, Richards, and logistic models, and coefficient values (*β0* = intercept, *β*1 = maximum relative growth rate, *β2* = upper asymptote, *β3* = weight loss begins*)* for the cubic polynomial model. We fitted the model to compare the first *vs.* second hatched nestlings and males *vs.* females. The analyses and figures were performed using GraphPad Prism Software, Inc. v. 8^38^.

## Results

### Body mass

**Figure 3:**
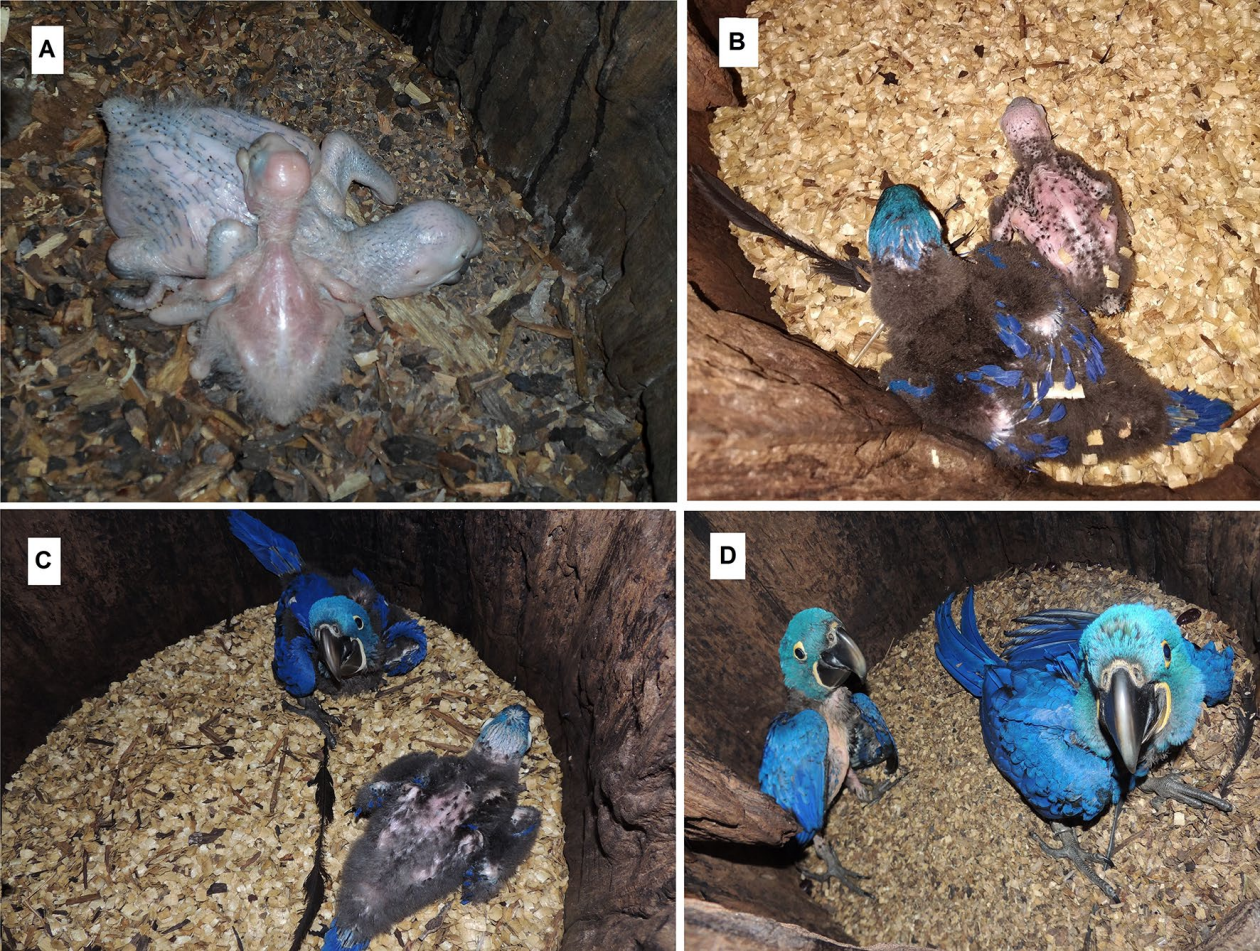
Development phases of the chicks: (**A**) nestling, 0–25 days, birds on picture are 17 and 18 days, respectively. Mass gain at this phase is slow, (**B**) chick, 26–77 days, birds on picture are 44 and 45 days, respectively, (**C**) chicks, birds on picture are 61 and 62 days, respectively. Geometric growth until maximum weight is attained, (**D**) juvenile, 78–107 days, birds on pictures are 104 and 105 days, respectively. Weight is maintained up to 90–95 days when weight loss begins with the first attempts of flying.

We weighed 42 eggs and obtained an average mass of 33.33 ± 3.99 g. Of these 30 normal-sized nestlings were monitored weekly at the same time of day. The average weight of the 30 nestlings was 23.00 ± 2.94 g/individual at day 1, 212.83 ± 32.62 g at day 15, 612.05 ± 30.47 g at day 30, 1188.74 ± 99.83 g at 60 days, and 1362.75 ± 47.10 g at 90 days. When the chicks left the nest after 107 days (average of 104 ± 4.4 days), their average weight was 1177.50 ± 105.47 g, while the maximum weight was 1550 g (average of 1240 ± 120 g). Chick development encompassed three phases: (1) nestling (0–25 days), during which chicks were completely dependent on their parents for maintaining body temperature, and weight gain was slow (Fig. 3A; nest with a normal-sized nestling and a dwarf one), (2) chick (26–77 days), a phase in which the mass gain was fast until maximum weight was reached (Fig. 3B = 44 and 45 days and Fig. 3C = 61 and 62 days), (3) youth (78–107 days), whereby weight was maintained up to 90–95 days (Fig. 3D), followed by weight loss concomitantly with the first attempts at flying and until permanently leaving the nest.

**Figure 4:**
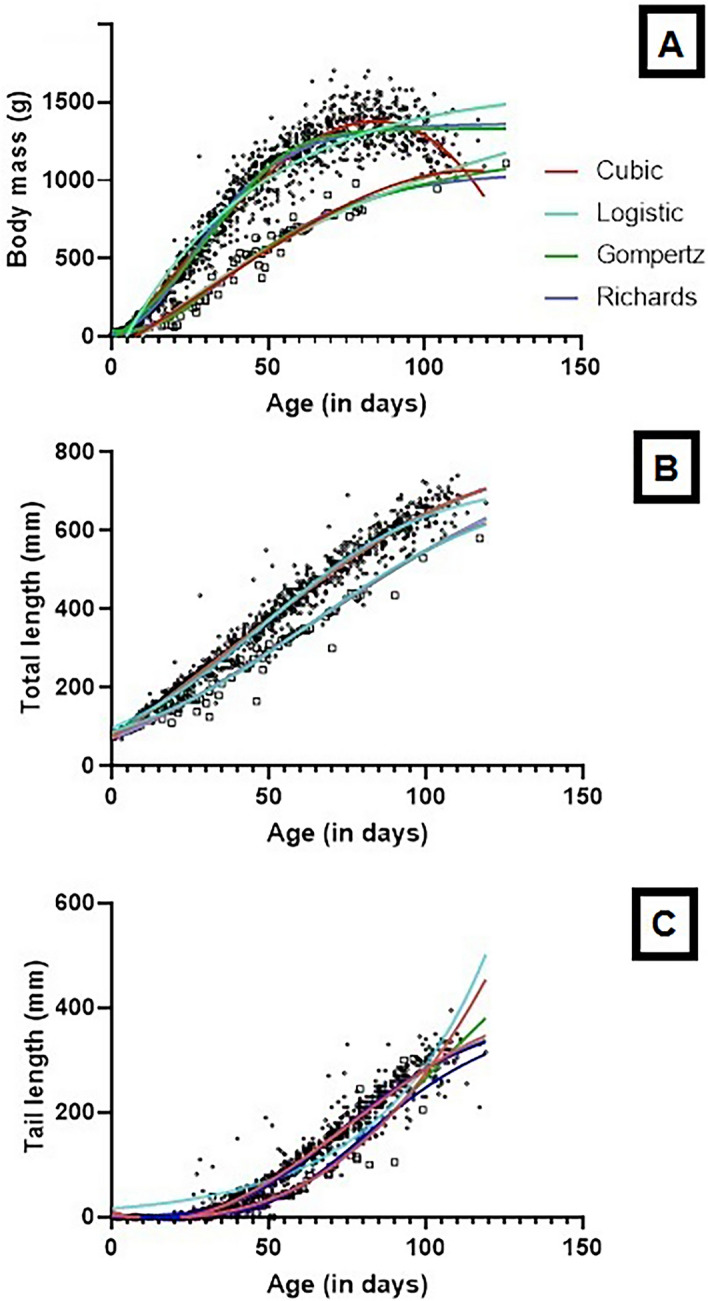
Growth curves fit of the four models tested in 412 nestlings of hyacinth macaws monitored over 30 years. The full black circles represent 381 individuals, and the empty squares represent the 15 dwarf individuals. (**A**) body mass, (**B**) total length, and (**C**) = tail length. Figures were produced using GraphPad Prism v. 8.

All models for normal-sized individual (n = 381) revealed similar R^2^ values for body mass with AIC indicating that the cubic polynomial performed best, probably because hyacinth macaw chicks lost weight during the final stages of development in the nest, and this loss required a more complex model (Table 1, Fig. 4A).

**Table 1:** Results from four models fitted to body mass growth to normal-sized and dwarfs hyacinth macaw.

Body mass normal-sized (g); n = 381							Body Mass-Dwarf (g); n = 15					F test; p value
Model	Parameters	Values	SE	R^2^	AIC	SE residual	Values	SE	R^2^	AICc	SE residual	
Gompertz	A	1363	7.819	0.948	11,295	110.8	1055	45.22	0.9564	648.4	58.51	334.5_(3, 1272)_; p < 0.0001
	Y0	18.56	2.466				14.10	4.803				
	*k*	0.0578	0.0011				0.039	0.0028				
Richards	A	1333	9.455	0.9491	11,272	109.7	1360	314.66	0.9578	650.4	58.72	4.0_(3, 1268)_; p = 0.007
	Y0	− 8.697	14.16				37.90	35.11				
	*k*	6.619	0.8129				1.554	0.862				
Logistic	A	1324	6.328	0.9468	11,322	112.1	954.0	59.42	0.9500	659.2	62.64	226.5_(3, 1272)_; p < 0.0001
	Y0	72.51	3.658				47.45	10.54				
	*k*	0.0884	0.0017				0.0668	0.0039				
Cubic polynomial	*β0*	− 65.25	10.37	0.9501	11,207	108.5	− 87.38	37.33	0.9499	661.6	63.11	269.8_(4, 1270)_; p < 0.0001
	*β1*	22.27	0.8749				11.49	2.516				
	*β2*	0.0796	0.0193				0.0593	0.047				
	*β3*	− 0.0016	0.0001				− 0.0006	0.0003				

Parameters of the Gompertz, Richards and Logistic models: *A* upper asymptote (i.e. predicted adult size), *k* maximum relative growth rate, *Y0* initial value of the asymptotic curve. Cubic polynomial models are represented with four parameters: *β0* intercept, *β1* maximum relative growth rate, *β2* upper asymptote, *β3* weight loss begins.

Field observations and cluster analysis showed that 15 individuals left the nest at a maximum of 126 days (average of 108 ± 15 days) with a maximum mass of 1000 g (average of 938 ± 63 g), which was approximately 22% less than that of the normal-sized individuals. This difference was reproduced by the models, with the asymptotes for the dwarfs reaching 77.4% of the Gompertz model, 72.2% of the logistic model, and 74.5% of the cubic polynomial model. Weight gain by the dwarfs was also lower compared to normal-sized birds, reaching 68.5% of the Gompertz model, 23.5% of the Richards model, 75.6% of the logistic model, and 51.6% of the cubic polynomial model (Table 1). When comparing the results generated with the Richards model, the differences were less pronounced, but the difference between the two groups remained significant (Table 1). Therefore, the dwarfs gained less weight (A) and did so more slowly (*k*). They reached maximum weight only when they were ready to leave the nest without any weight loss prior to nest abandonment, which resulted in the lowest AIC for the Gompertz model (Table 1, Fig. 4A).

### Total length

The average length and width of a hyacinth macaw egg (n = 42) was 47.32 ± 0.96 mm and 36.59 ± 0.93 mm, respectively. The 30 chicks monitored with high-resolution temporal measured on average 77.00 ± 1.00 mm and had a total length of 147.00 ± 11.64 mm at day 15, 249.00 ± 13.46 mm at day 30, 417.12 ± 7.52 mm at day 60, and 591.09 ± 53.81 mm at day 90. When leaving the nest, the average total length was 677.25 ± 12.91 mm. Concerning the 381 normal-sized individuals, the Richards and cubic polynomial models performed best in describing total length, i.e. these models had the lowest AIC (Table 2). Both models showed a predominance of the linear phase, with *k* = 7.79 (Richards) and *β1* = 5.26 (cubic polynomial, Table 2; Fig. 4B). When total length curves were compared, significant differences were observed between the normal-sized individuals and the dwarfs. The latter not described well by the Richards model, with large standard error values denoting uncertainty in the model’s fit. The Gompertz and logistic models exhibited the best performance based on AIC, and, despite the low value of the asymptote, the dwarfs maintained the same rate of growth (*k*) compare to total length normal-sized birds in the logistic model (Table 2).

**Table 2:** Results from four models fitted to total length growth to normal-sized and dwarfs hyacinth macaw.

Total length normal-sized (mm); n = 381							Total length (mm) Dwarf; n = 15					F test; p value
Model	Parameters	Values	SE	R^2^	AIC	SE Residual	Values	SE	R^2^	AICc	SE Residual	
Gompertz	*A*	856.4	12.41	0.9734	8080	29.75	755.4	118.08	0.9550	436.4	21.71	108.8_(3, 1256)_; p < 0.0001
	*Y0*	82.75	1.682				72.17	7.032				
	*k*	0.0207	0.0004				0.0185	0.003				
Richards	*A*	717.0	31.02	0.9739	8063	29.51	701.3	*	0.9642	489.2	25.81	–
	*Y0*	69.39	3.585				82.98					
	*k*	7.795	2.252				2.766					
Logistic	*A*	722.9	6.358	0.9713	8173	30.93	559.6	43.11	0.9547	436.8	21.76	97.76_(3, 1256)_; p < 0.0001
	*Y0*	97.35	1.509				80.53	5.684				
	*k*	0.0383	0.0005				0.0384	0.003				
Cubic polynomial	*β0*	72.75	2.867	0.9738	8063	29.53	76.49	16.66	0.9549	438.8	21.88	84.48_(4, 1254)_; p < 0.0001
	*β1*	5.258	0.2424				2.727	1.475				
	*β2*	0.0262	0.0053				0.0528	0.038				
	*β3*	− 0.0002	0.0003				− 0.0003	0.0003				

Parameters of the Gompertz, Richards and Logistic models: *A* upper asymptote (i.e. predicted adult size), *k* maximum relative growth rate, *Y0* initial value of the asymptotic curve. Cubic polynomial models are represented with four parameters: *β0* intercept, *β1* maximum relative growth rate, *β2* upper asymptote, *β3* weight loss begins.

*Very wide confidence interval = CI > 95%.

### Tail length

In general, the tail feathers grew rather rapidly. The first pin feathers appeared after 25–31 days and, by the time the birds left the nest, the tail feathers accounted for approximately 50% of the total length of the tail. Although the Richards model was numerically better and Gompertz was the second best model for the 381 normal-sized individuals (Table 3; Fig. 4C). The tail feathers of 15 dwarf birds were best described by a Gompertz model, which resulted in dwarfs with total length, on average, 70% smaller and asymptote 84% higher for the dwarfs than the normal-sized chicks. This indicates either an overestimation or that the dwarfs left the nest during the linear growth phase before reaching the asymptote. In the Gompertz model, the growth rate (*k*) was 30% slower, and explained 93.9% of the variation observed in these individuals. The other models did not fit the tail growth data as in the case of the Richards and Logistic models, resulting in wide confidence intervals (CI > 95%) which results in model instability.

**Table 3:** Results from four models fitted to tail length growth to normal-sized and dwarfs hyacinth macaw.

Tail length normal-sized (mm); n = 381							Tail length (mm) Dwarf; n = 15					F test; p value
Model	Parameters	Values	SE	R^2^	AIC	SE Residual	Values	SE	R^2^	AICc	SE Residual	
Gompertz	A	425.3	15.91	0.9324	7648	26.14	2801	2429	0.9389	405.8	16.12	23.97_(3, 1238)_; p < 0.0001
	Y0	0.0689	0.0337				0.0955	0.1419				
	*k*	0.0303	0.0013				0.0162	0.0046				
Richards	A	428.4	90.11	0.9570	7103	20.86	302.1	*	0.8517	511.1	29.97	–
	Y0	0.001	1.711				2.059					
	*k*	3.663	0.9269				98.90					
Logistic	A	328.3	6.233	0.9295	7697	37.34	~ 0.0000		0.9243	421.3	17.94	–
	Y0	2.782	0.2877				2.803	*				
	*k*	0.0650	0.0017				0.0500					
Cubic polynomial	*β0*	11.76	1.989	0.9317	7662	26.28	− 4.529	9.196	0.9382	409	16.33	27.53_(4, 1231)_; p < 0.0001
	*β1*	− 2.449	0.1668				0.7091	0.7671				
	*β2*	0.091	0.0037				− 0.0317	0.0182				
	*β3*	− 0.0003	0.0000				0.0006	0.0001				

Parameters of the Gompertz, Richards and Logistic models: *A* upper asymptote (i.e. predicted adult size), *k* maximum relative growth rate, *Y0* initial value of the asymptotic curve. Cubic polynomial models are represented with four parameters: *β0* intercept, *β1* maximum relative growth rate, *β2* upper asymptote, *β3* weight loss begins.

*Very wide confidence interval = CI > 95%.

### Differences among nestlings

**Figure 5:**
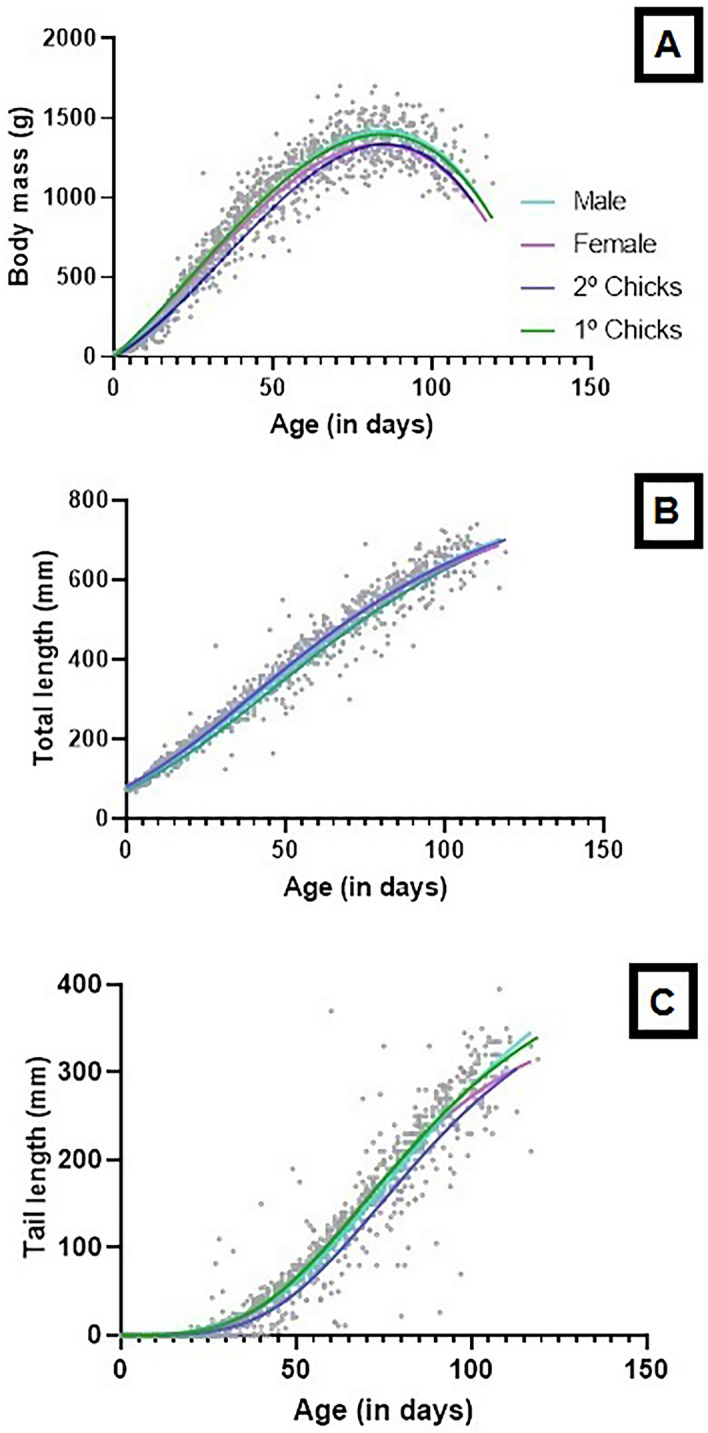
Fit of growth curves to 1st chick and 2nd chick, and to male and female hyacinth macaws. (**A**) = body mass; cubic polynomial model, (**B**) = total length; Gompertz model and (**C**) = tail length; Gompertz model. Figures were produced using GraphPad Prism v. 8.

We used the cubic polynomial and Gompertz models to compare the first (n = 294) and second hatched (n = 87) chicks as well as males (n = 148) and females (n = 226), all of which were normal-sized (Fig. 5A–C). This analysis revealed differences between model parameters (Table 4). Weight gain was slower for the second chick (*β1* = 13.51) and 42% faster for the first chick during the linear phase (*β1* = 23.29), this result reflected a significant difference in the fitting of the curves. Nevertheless, the goodness of fit was significantly different, being male model better than female. The values obtained for the asymptote and rate of increase in total length for the first and second chicks were statistically different. For the mass increment, the variation to model parameters was not so clear, with *β0, β1, β3* without differences and for *β1* the difference was significant between males and females. In addition, the AIC values indicated that the curve fitted better to the male chicks dataset. Total growth between sex not was statistically different, including the curve fit for the two datasets, confirming the lack of sexual dimorphism in total length (Table 4; Fig. 5B). However, there were differences between tail size, with tail size 24% greater in males than in females (Fig. 5C).

**Table 4:** Parameter results from two models fitted to growth of first and second chicks, male, and female of normal-sized hyacinth macaws.

Traits	1st chick (n = 294)				2nd chick (n = 87)				Male (n = 148)			Female (226)			
		Values	R^2^	AIC	Values	R^2^	AIC	F test; p value	Values	R^2^	AIC	Values,	R^2^	AIC	F test; p value
Body mass (g)	*β0*	− 71.43^a^	0.953	8883	− 24.01^b^	0.965	6815	29.29_(4, 1198)_; p < 0.0001	− 65.2^a^	0.948	3407	− 73.53^a^	0.952	3711	12.52_(4, 760)_; p < 0.0001
	*β1*	23.29^a^			13.51^b^				21.54^a^			22.94^a^			
	*β2*	0.063^a^			0.242^b^				0.109^a^			0.053^b^			
	*β3*	− 0.0016^a^			− 0.0025^b^				− 0.0018^a^			− 0.0015^a^			
Total length (mm)	A	846.8^a^	0.968	6577	863.2^a^	0.963	1779	24.34_(3, 1192)_; p < 0.0001	868.2^a^	0.969	2518	819.2^a^	0.953	2957	1.313_(3, 763)_; p = 0.2689
	Y0	83.49^a^			74.00^b^				80.35^a^			80.14^a^			
	*k*	0.0211^a^			0.0202^a^				0.0207^a^			0.0221^a^			
Tail (mm)	A	429.5^a^	0.948	5895	400.5^a^	0.904	1612	27.75_(3, 1173)_; p < 0.0001	487.8^a^	0.932	2382	372^b^	0.930	2626	4.110_(3, 757)_; p = 0.0066
	Y0	0.077^a^			0.016^a^				0.187^a^			0.023^a^			
	*k*	0.030^a^			0.031^a^				0.027^a^			0.034^b^			

Cubic polynomial model (*β0* = intercept, *β*1 = maximum relative growth rate, *β2* = upper asymptote, *β3* = weight loss begins) and Gompertz model (A = upper asymptote, i.e. predicted adult size), *k* maximum relative growth rate, *Y0* initial value).

*Values follow of different letters are significant to p < 0.05.

## Discussion

The total period of development of the hyacinth macaw normal-sized chicks lasted 107 days with an average final body mass of 1177.5 ± 105.4 g. Given that this is the largest flying parrot, hole-nesting, feature small clutches, and a specialized diet, is expected to have slower growth rate than for other species^34^, such as *Calyptorhynchus latirostris*^39^, *Myiopsitta monachus*^40^, *Ara ararauna*^41^, *Forpus passerinus*^42^, *Amazona autumnalis,* *Amazona* *oratrix*, *Amazona viridigenalis*^43^, *Primolius maracana*^44^, *Cyanoliseus patagonus*^20^, *Amazona aestiva*^45^, and *Ara macao*^46^.

The AIC values showed that the choice of model depended on the growth metrics analyzed. Accordingly, the best models for normal-sized individuals were the cubic polynomial for body mass, Richards and cubic polynomial for total length, and Gompertz for tail length. Tjørve and Tjørve^47^ comment that species with growth patterns that demand more complex growth models are quite uncommon. The post-natal growth rate results in hyacinth macaws to reach adult body mass late in the growth cycle, that is, after abandoning the nest but while still needing parental care^48–50^. This phenomenon is observed also in species whose young reach peak body mass before starting flight exercises, which results in weight loss while still in the nest, mass recession, and the attainment of adult body mass only after leaving the nest^51,52^. In these instances, the Gompertz, Richards, and logistic growth curves, can yield unrealistic values. This was the case for the hyacinth macaw, whose maximum weight was reached at approximately 80 days, but then declined before finally attaining adult weight after fledging. These behaviour help explain why the cubic polynomial model is the best fit for body mass; whereas the sigmoidal models is the best fit for total length and tail length, as its function better reflects the continuous growth of tail feathers until adult size is attained.

Our results corroborate the field observations and reveal significant intraspecific differences among growth of hyacinth macaw chicks. Many studies report the existence of intraspecific variation in growth rates, most of which is explained by the availability of food resources, physiological differences, low reproductive performance of parents, phenotypic plasticity, natural and anthropic environmental factors^17,20,53^. However, intraspecific variation related to growth rates can vary between species, principally between those that are altricial, wild, and display slow growth rates, which makes them more prone to suffer from environmental changes and predation^16,34,54,55^. These sources of variation, coupled with the large number of individuals sampled over a long period, had a strong influence on how the growth models fitted each biometric trait. For example, the wide range of initial values and inflection points determines more or less elongated curves and modifies the maximum asymptote, which in turn leads to individual body mass and total length variation and a growth rate slower or faster increase^17,47^. These sources of variation in birds are observed mainly as a function of the time it takes to hatch, sex, sampling year, and locality^16,19,20,54^.

Despite phenotypic variations and changes affecting the shape of the growth curve, we did not find studies describing different growth patterns within the same species, clutch, or locality in natural populations. Our results indicate that there are chicks observed in the natural habitat, which were much smaller than the rest of the evaluated chicks, thus confirming the supposed existence of dwarfs. In this dwarf group, body mass increment and overall weight gain were 54% (average of all models) below normal-sized and there was no weight loss before the birds left the nest. According to Ricklefs^16^, growth traits can exhibit as much as 20% intraspecific variation with respect to geographic location and time of the nesting season. In this context, all models revealed differences of less than 20% for the dwarfs, being approximately 22%, 27% and 70% to mass, total length, and tail length respectively. It is important to emphasize that these individuals will not attain the same adult size as the rest of the chicks and will always be easily recognizable in the field. Additionally, these individuals form couples with larger adults of both sexes and are not limited by low reproductive success^56^. These cases were identified due to the long-term design of this study, which involved 30 years of morphometric monitoring of free-living hyacinth macaws. As they represented only 8.1% of all measured individuals and that fledged, it would have been unlikely to identify dwarf individuals in short-term studies.

The growth models for the hyacinth macaw developed in this study demonstrate the specific and complex biological and behavioural characteristics of this species. Intraspecific variation, such as that between males and females and the first and second hatched nestlings, was observed. However, these differences can hardly be recognized by an observer in the field. On the other hand, dwarf animals can easily be recognized by an observer in the field, although most of the time they are confused with a young individual. Perhaps for this reason dwarfs have rarely been described in natural populations. Our finding opens opportunities to examine whether this species may take advantage of intraspecific variability to overcome difficulties such as the recent fires that reduced food availability. We believe that these results can also support the management of other Psittaciformes. For example, carrying out projects that identify the health of individuals which do not reach the maximum standard of growth, which are mainly those coming from voluntary surrender or are rescued. In addition, our results reaffirm the importance of long-term studies, especially for species that are vulnerable and threatened with extinction.

## Data Availability

The data is available to any personnel who wish to analyze any pattern or hypothesis. As long as you take into account the authorship of the database.
